# Supplementary material for: Phylogeny of the damselfishes (Pomacentridae) and patterns of asymmetrical diversification in body size and feeding ecology
Source: PLoS One. 2021 Oct 27;16(10):e0258889. doi: 10.1371/journal.pone.0258889 (PMC8550381; doi:10.1371/journal.pone.0258889)
Supplement: S1 File — (ZIP) [file pone.0258889.s001.zip › SupportingInfoFinal/S1_Fig_BestML.pdf]

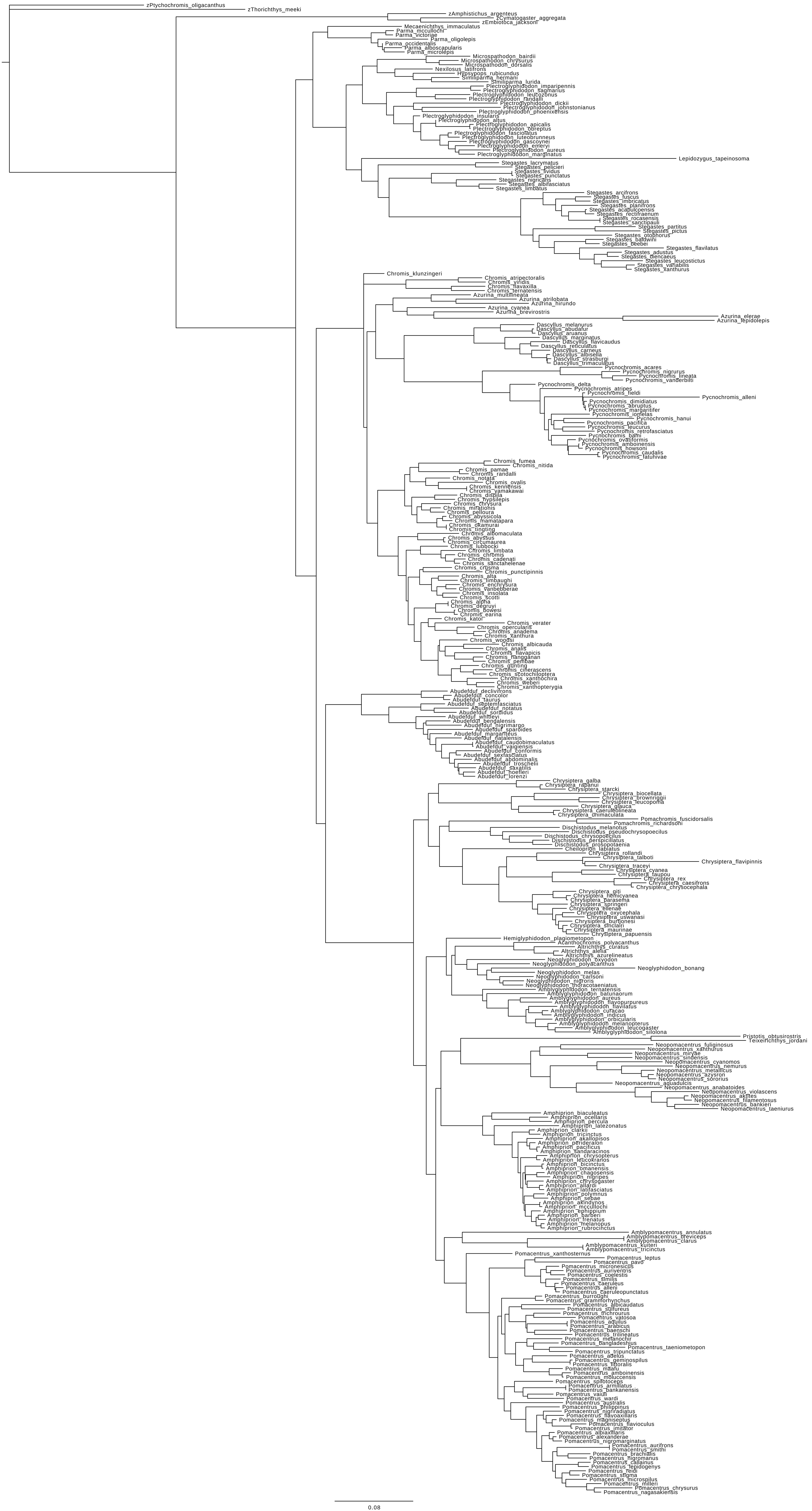
zPtychochromis_oligacanthus
zThorichthys_meeki
zAmphistichus_argenteus
zCymatogaster_aggregata
zEmbiotoca_jacksoni
Mecaenichthys_immaculatus
Parma_mccullochi
Parma_victoriae
Parma_oligolepis
Parma_occidentalis
Parma_alboscapularis
Parma_microlepis
Microspathodon_bairdii
Microspathodon_chrysurus
Microspathodon_dorsalis
Nexilosus_latifrons
Hypsypops_rubicundus
Similiparma_hermani
Similiparma_lurida
Plectroglyphidodon_imparipennis
Plectroglyphidodon_sagmarius
Plectroglyphidodon_leucozonus
Plectroglyphidodon_randalli
Plectroglyphidodon_dickii
Plectroglyphidodon_johnstonianus
Plectroglyphidodon_phoenixensis
Plectroglyphidodon_insularis
Plectroglyphidodon_altus
Plectroglyphidodon_apicalis
Plectroglyphidodon_obreptus
Plectroglyphidodon_fasciolatus
Plectroglyphidodon_luteobrunneus
Plectroglyphidodon_gascoynei
Plectroglyphidodon_emeryi
Plectroglyphidodon_aureus
Plectroglyphidodon_marginatus
Lepidozygus_tapeinosoma
Stegastes_lacrymatus
Stegastes_pelicieri
Stegastes_lividus
Stegastes_punctatus
Stegastes_nigricans
Stegastes_albifasciatus
Stegastes_limbatus
Stegastes_arcifrons
Stegastes_fuscus
Stegastes_imbricatus
Stegastes_planifrons
Stegastes_acapulcoensis
Stegastes_rectifraenum
Stegastes_rocasensis
Stegastes_sanctipauli
Stegastes_partitus
Stegastes_pictus
Stegastes_otophorus
Stegastes_baldwini
Stegastes_beebei
Stegastes_flavilatus
Stegastes_adustus
Stegastes_diencaeus
Stegastes_leucostictus
Stegastes_variabilis
Stegastes_xanthurus
Chromis_klunzingeri
Chromis_atripectoralis
Chromis_viridis
Chromis_flavaxilla
Chromis_ternatensis
Azurina_multilineata
Azurina_atrilobata
Azurina_hirundo
Azurina_cyanea
Azurina_brevirostris
Azurina_elerae
Azurina_lepidolepis
Dascyllus_melanurus
Dascyllus_abudafur
Dascyllus_aruanus
Dascyllus_flavicaudus
Dascyllus_marginatus
Dascyllus_reticulatus
Dascyllus_carneus
Dascyllus_albisella
Dascyllus_strasburgi
Dascyllus_trimaculatus
Pycnochromis_acares
Pycnochromis_nigrurus
Pycnochromis_lineata
Pycnochromis_vanderbilti
Pycnochromis_delta
Pycnochromis_atripes
Pycnochromis_fieldi
Pycnochromis_alleni
Pycnochromis_dimidiatus
Pycnochromis_abruptus
Pycnochromis_margaritifer
Pycnochromis_iomelas
Pycnochromis_hanui
Pycnochromis_pacifica
Pycnochromis_leucurus
Pycnochromis_retrofasciatus
Pycnochromis_bami
Pycnochromis_ovatiformis
Pycnochromis_amboinensis
Pycnochromis_howsoni
Pycnochromis_caudalis
Pycnochromis_latuhivae
Chromis_fumea
Chromis_nitida
Chromis_pamae
Chromis_randalli
Chromis_notata
Chromis_ovalis
Chromis_kennensis
Chromis_yamakawai
Chromis_dispila
Chromis_hypsilepis
Chromis_chrysura
Chromis_mirationis
Chromis_pelloura
Chromis_abyssicola
Chromis_mamatapara
Chromis_okamurai
Chromis_tingting
Chromis_albomaculata
Chromis_abyssus
Chromis_circumaurea
Chromis_lubbocki
Chromis_limbata
Chromis_chromis
Chromis_cadenati
Chromis_sanctahelenae
Chromis_crusma
Chromis_punctipinnis
Chromis_alta
Chromis_limbaughi
Chromis_enchrysura
Chromis_vanbebberae
Chromis_insolata
Chromis_scotti
Chromis_alpha
Chromis_degruyi
Chromis_bowesi
Chromis_earina
Chromis_kator
Chromis_verater
Chromis_opercularis
Chromis_anadema
Chromis_xanthura
Chromis_woodsi
Chromis_albicauda
Chromis_analis
Chromis_flavapicis
Chromis_hangganan
Chromis_pembae
Chromis_gunting
Chromis_cinerascens
Chromis_scotochiloptera
Chromis_xanthochira
Chromis_weberi
Chromis_xanthopterygia
Abudefduf_declivifrons
Abudefduf_concolor
Abudefduf_taurus
Abudefduf_septemfasciatus
Abudefduf_notatus
Abudefduf_sordidus
Abudefduf_whitleyi
Abudefduf_bengalensis
Abudefduf_nigrimargo
Abudefduf_sparoides
Abudefduf_margariteus
Abudefduf_natalensis
Abudefduf_caudobimaculatus
Abudefduf_vaigiensis
Abudefduf_conformis
Abudefduf_sexfasciatus
Abudefduf_abdominalis
Abudefduf_troschelii
Abudefduf_saxatilis
Abudefduf_hoefleri
Abudefduf_lorenzi
Chrysiptera_galba
Chrysiptera_rapanui
Chrysiptera_starcki
Chrysiptera_biocellata
Chrysiptera_brownriggii
Chrysiptera_leucopoma
Chrysiptera_glauca
Chrysiptera_caeruleolineata
Chrysiptera_unimaculata
Pomachromis_fuscidorsalis
Pomachromis_richardsoni
Dischistodus_melanotus
Dischistodus_pseudochrysopoecilus
Dischistodus_chrysopoecilus
Dischistodus_perspicillatus
Dischistodus_prosopotaenia
Cheiloprion_labiatus
Chrysiptera_rollandi
Chrysiptera_talboti
Chrysiptera_flavipinnis
Chrysiptera_traceyi
Chrysiptera_cyanea
Chrysiptera_taupou
Chrysiptera_rex
Chrysiptera_caesifrons
Chrysiptera_chrysocephala
Chrysiptera_giti
Chrysiptera_hemicyanea
Chrysiptera_parasema
Chrysiptera_springeri
Chrysiptera_ellenae
Chrysiptera_oxycephala
Chrysiptera_uswanasi
Chrysiptera_burtjonesi
Chrysiptera_sinclairi
Chrysiptera_maurinae
Chrysiptera_papuensis
Hemiglyphidodon_plagiometopon
Acanthochromis_polyacanthus
Altrichthys_curatus
Altrichthys_alelia
Altrichthys_azurelineatus
Neoglyphidodon_oxyodon
Neoglyphidodon_polyacanthus
Neoglyphidodon_bonang
Neoglyphidodon_melas
Neoglyphidodon_carlsoni
Neoglyphidodon_nigroris
Neoglyphidodon_thoracotaeniatus
Amblyglyphidodon_ternatensis
Amblyglyphidodon_batunaorum
Amblyglyphidodon_aureus
Amblyglyphidodon_flavopurpureus
Amblyglyphidodon_flavilatus
Amblyglyphidodon_curacao
Amblyglyphidodon_indicus
Amblyglyphidodon_orbicularis
Amblyglyphidodon_melanopterus
Amblyglyphidodon_leucogaster
Amblyglyphidodon_silolona
Pristotis_obtusirostris
Teixeirichthys_jordani
Neopomacentrus_fuliginosus
Neopomacentrus_xanthurus
Neopomacentrus_miryae
Neopomacentrus_sindensis
Neopomacentrus_cyanomos
Neopomacentrus_nemurus
Neopomacentrus_metallicus
Neopomacentrus_azysron
Neopomacentrus_sororius
Neopomacentrus_aquadulcis
Neopomacentrus_anabatoides
Neopomacentrus_violascens
Neopomacentrus_aktites
Neopomacentrus_filamentosus
Neopomacentrus_bankieri
Neopomacentrus_taeniurus
Amphiprion_biaculeatus
Amphiprion_ocellaris
Amphiprion_percula
Amphiprion_latezonatus
Amphiprion_clarkii
Amphiprion_tricinctus
Amphiprion_akallopisos
Amphiprion_perideraion
Amphiprion_pacificus
Amphiprion_sandaracinos
Amphiprion_chrysopterus
Amphiprion_leucokranos
Amphiprion_bicinctus
Amphiprion_omanensis
Amphiprion_chagosensis
Amphiprion_nigripes
Amphiprion_chrysogaster
Amphiprion_allardi
Amphiprion_latifasciatus
Amphiprion_polymnus
Amphiprion_sebae
Amphiprion_akindynos
Amphiprion_mccullochi
Amphiprion_ephippium
Amphiprion_barberi
Amphiprion_frenatus
Amphiprion_melanopus
Amphiprion_rubrocinctus
Amblypomacentrus_annulatus
Amblypomacentrus_breviceps
Amblypomacentrus_clarus
Amblypomacentrus_kuiteri
Amblypomacentrus_tricinctus
Pomacentrus_xanthosternus
Pomacentrus_leptus
Pomacentrus_pavo
Pomacentrus_micronesicus
Pomacentrus_auriventris
Pomacentrus_coelestis
Pomacentrus_similis
Pomacentrus_caeruleus
Pomacentrus_alleni
Pomacentrus_caeruleopunctatus
Pomacentrus_burroughi
Pomacentrus_grammorhynchus
Pomacentrus_albicaudatus
Pomacentrus_sulfureus
Pomacentrus_trichrourus
Pomacentrus_vatosoa
Pomacentrus_aquilus
Pomacentrus_arabicus
Pomacentrus_baenschi
Pomacentrus_trilineatus
Pomacentrus_melanochir
Pomacentrus_bangladeshius
Pomacentrus_taeniometopon
Pomacentrus_tripunctatus
Pomacentrus_adelus
Pomacentrus_geminospilus
Pomacentrus_littoralis
Pomacentrus_maafu
Pomacentrus_amboinensis
Pomacentrus_moluccensis
Pomacentrus_spilotoceps
Pomacentrus_armillatus
Pomacentrus_bankanensis
Pomacentrus_vaiuli
Pomacentrus_wardi
Pomacentrus_australis
Pomacentrus_philippinus
Pomacentrus_nigriradiatus
Pomacentrus_flavoaxillaris
Pomacentrus_magniseptus
Pomacentrus_flavioculus
Pomacentrus_imitator
Pomacentrus_albiaxillaris
Pomacentrus_alexanderae
Pomacentrus_nigromarginatus
Pomacentrus_aurifrons
Pomacentrus_smithi
Pomacentrus_brachialis
Pomacentrus_nigromanus
Pomacentrus_callainus
Pomacentrus_lepidogenys
Pomacentrus_reidi
Pomacentrus_stigma
Pomacentrus_microspilus
Pomacentrus_milleri
Pomacentrus_chrysurus
Pomacentrus_nagasakiensis
0.08
