# Supplementary material for: Phylogeny of the damselfishes (Pomacentridae) and patterns of asymmetrical diversification in body size and feeding ecology
Source: PLoS One. 2021 Oct 27;16(10):e0258889. doi: 10.1371/journal.pone.0258889 (PMC8550381; doi:10.1371/journal.pone.0258889)
Supplement: S1 File — (ZIP) [file pone.0258889.s001.zip › SupportingInfoFinal/S1_Text_ Methods.docx]

**Supplemental Methods**

**DNA sequencing**

DNA was extracted from muscle or gill tissue according to the PureGene animal tissue DNA isolation procedure (Gentra System). Double-stranded DNA products were amplified using polymerase chain reaction from aliquots of genomic DNA isolates of the 12S, 16S, ND3, tmo4c4, rag1, rag2, bmp4 and DLX2 gene fragments. Primers used for DNA amplification and sequencing are listed in Supplemental Table 2. PCR reactions were performed using an MJ Research PTC-200 Peltier Thermal Cycler (MJ Research Inc., Watertown, MA). PCR products were cycle-sequenced by creating a solution of 3.3 ul of buffer, 0.5 ul of primer, 0.7 ul of Big Dye reagent, 0.5 ul of PCR product template DNA and 5.0 ul nuclease free water to bring the total reaction volume to 10 ul. Thermal cycling protocol included one initial denaturation at 96° C followed by 25 cycles of denaturation at 96° C for 10 s, annealing at 50° C for 5 s and extension at 60° C for 4 min. All products were purified and sequences were generated on an ABI PRISM 3730 Genetic Analyzer (Applied Biosystem, Foster City, CA). All raw sequence data have been deposited in GenBank (see Table S1 for accession numbers).

Sequences for 12s and 16s were manually aligned to secondary structure models for ribosomal sequences using previously published homologous sequences for damselfishes and outgroups (1) in Mesquite version 3.51 (53). Protein coding genes were automatically aligned using the ClustalW or Muscle modules within Mesquite (54,55). Some sequences were trimmed to the size of the smallest fragment to minimize the amount of missing data in the data matrix. After excluding unalignable regions and gaps, the character count for the entire concatenated supermatrix was 8238 nucleotide characters.

**Matrix construction and alignment: comparison with the recent Tang et al. 2021 analysis.**

The present study was released in preprint shortly before the Tang et. al (37) study was published. In review this enabled us to adopt the much-needed taxonomic revisions provided therein, and to incorporate some of the new sequence data generated by that study. A large portion of the data matrices used in our study and the Tang et al. 2021 study are the same, often the exact same sequences assembled from our own and each other’s work, and that of colleagues over the last 20 years. However, some of the sequences are the same locus, same species, but different individuals. Also, we assembled a set of 12 genes for 345 taxa, to build on prior work and attempt to achieve as close as possible to a total evidence phylogenetic tree.

Importantly, the Tang et. al study (37) did a great community service by sleuthing out the various specimen identification errors or contamination problems that inevitably creep into these large data depositions, and we have revised our matrix accordingly to adjust specimen identifications and remove erroneous sequences. Some additional notes and differences in matrix construction:

1. Tang et al. used the Histone3 locus, which we don’t have and decided not to adopt in our final analysis. We noted that the H3 data has a fairly high frequency of ambiguous base calls, and covered 331 bases for 151 damselfishes, so opted not to include it in our analysis. We also found and alerted the authors that the H3 sequence for one species (*Chromis chromis*) was reverse complemented in the final matrix, apparently without a significant impact on their topology (Tang, pers. comm.).

2. The mitochondrial large and small subunit ribosomal RNA loci 12S and 16S are typically informative at multiple levels for damselfish phylogeny, and the treatment of these sequences was different in our studies. We used secondary RNA structure models of the stems (which complement another stem downstream) and loops (which do not complement downstream code) to do the initial alignments, with a serial process of segment identification and auto alignment of loops only using Muscle and/or Clustal. The entire sequences for 12S and 16S rRNA were aligned by MAFFT for the Tang et al. analysis, and it does appear that some features of the secondary structure of the alignment are lost thereby.

3. Anomalous alignments and insertions are also noted in the published rRNA sequences for the Tang et al. 2021 matrix, including large insertions in the 12S seq of bases 385-409 for *Stegastes partitus* and a large insertion which appears to be a duplication of downstream sequence from position 601-708 for *Amphiprion ephippium*. In addition, the first 5-6 bases of the 16S sequence starting at position 1151 also appear to be misaligned for numerous taxa.

**Hidden-State Model Testing**

We used a multistate, hidden-state speciation and extinction (MuHiSSE) model framework (66) to evaluate the association of transitions among body size categories and dietary ecotypes with rates of diversification across the damselfish phylogeny. All analyses were performed using the hisse package in R (66). We generated eight models to test hypotheses related to our focal traits of interest (size and ecotype), their association with diversification, and patterns of transitions among states. Model one assumes a single rate of turnover and extinction fraction for the entire phylogeny. Turnover and extinction fraction were allowed to vary with the focal trait in models 2 through 6. Hidden states were included in models 3 through 8. Transitions between hidden states were set at a constant rate, with the exception of Model 4 (MuHiSSE Relaxed), in which we allowed for variation in the directionality of transitions between each hidden state. Models five and six test the evolutionary dead-end hypothesis by setting transitions to the non-intermediate states to zero. We used both a two and three state character independent model (CID2 and CID3), in which diversification parameters are associated with hidden character states, as our null models to assess the true effect of our focal trait on variation in tip associated diversification rates. The schematics used to parameterize each model are shown below, with each free parameter associated with diversification and transitions indicated by a unique number.

Model Parameterization Setup

Model 1: “Dull” Model

Diversification Parameters:

|  | Small | Medium | Large |
| --- | --- | --- | --- |
| Turnover | 1 | 1 | 1 |
| Extinction Fraction | 1 | 1 | 1 |

Transition Parameters:

|  | Small | Medium | Large |
| --- | --- | --- | --- |
| Small | - | 3 | 5 |
| Medium | 1 | - | 6 |
| Large | 2 | 4 | - |

Model 2: MuSSE

Diversification Parameters:

|  | Small | Medium | Large |
| --- | --- | --- | --- |
| Turnover | 1 | 2 | 3 |
| Extinction Fraction | 4 | 5 | 6 |

Transition Parameters:

|  | Small | Medium | Large |
| --- | --- | --- | --- |
| Small | - | 3 | 5 |
| Medium | 1 | - | 6 |
| Large | 2 | 4 | - |

Model 3: MuHiSSE Model

Diversification Parameters:

|  | Small A | Medium A | Large A | Small B | Medium B | Large B |
| --- | --- | --- | --- | --- | --- | --- |
| Turnover | 1 | 2 | 3 | 4 | 5 | 6 |
| Extinction Fraction | 7 | 8 | 9 | 10 | 11 | 12 |

Transition Parameters:

|  | Small A | Medium A | Large A | Small B | Medium B | Large B |
| --- | --- | --- | --- | --- | --- | --- |
| Small A | - | 3 | 5 | 13 | - | - |
| Medium A | 1 | - | 6 | - | 13 | - |
| Large A | 2 | 4 | - | - | - | 13 |
| Small B | 13 | - | - | - | 9 | 11 |
| Medium B | - | 13 | - | 7 | - | 12 |
| Large B | - | - | 13 | 8 | 10 | - |

Model 4: MuHiSSE Model (Relaxed)

Diversification Parameters:

|  | Small A | Medium A | Large A | Small B | Medium B | Large B |
| --- | --- | --- | --- | --- | --- | --- |
| Turnover | 1 | 2 | 3 | 4 | 5 | 6 |
| Extinction Fraction | 7 | 8 | 9 | 10 | 11 | 12 |

Transition Parameters:

|  | Small A | Medium A | Large A | Small B | Medium B | Large B |
| --- | --- | --- | --- | --- | --- | --- |
| Small A | - | 3 | 5 | 14 | - | - |
| Medium A | 1 | - | 6 | - | 14 | - |
| Large A | 2 | 4 | - | - | - | 14 |
| Small B | 13 | - | - | - | 9 | 11 |
| Medium B | - | 13 | - | 7 | - | 12 |
| Large B | - | - | 13 | 8 | 10 | - |

Model 5: Small/Benthic Absorbing

Diversification Parameters:

|  | Small A | Medium A | Large A | Small B | Medium B | Large B |
| --- | --- | --- | --- | --- | --- | --- |
| Turnover | 1 | 2 | 3 | 4 | 5 | 6 |
| Extinction Fraction | 7 | 8 | 9 | 10 | 11 | 12 |

Transition Parameters:

|  | Small A | Medium A | Large A | Small B | Medium B | Large B |
| --- | --- | --- | --- | --- | --- | --- |
| Small A | - | 1 | 3 | 9 | - | - |
| Medium A | 0 | - | 4 | - | 9 | - |
| Large A | 0 | 2 | - | - | - | 9 |
| Small B | 9 | - | - | - | 0 | 0 |
| Medium B | - | 9 | - | 5 | - | 8 |
| Large B | - | - | 9 | 6 | 7 | - |

Model 6: Large/Pelagic Absorbing

Diversification Parameters:

|  | Small A | Medium A | Large A | Small B | Medium B | Large B |
| --- | --- | --- | --- | --- | --- | --- |
| Turnover | 1 | 2 | 3 | 4 | 5 | 6 |
| Extinction Fraction | 7 | 8 | 9 | 10 | 11 | 12 |

Transition Parameters:

|  | Small A | Medium A | Large A | Small B | Medium B | Large B |
| --- | --- | --- | --- | --- | --- | --- |
| Small A | - | 2 | 3 | 9 | - | - |
| Medium A | 1 | - | 4 | - | 9 | - |
| Large A | 0 | 0 | - | - | - | 9 |
| Small B | 9 | - | - | - | 6 | 7 |
| Medium B | - | 9 | - | 5 | - | 8 |
| Large B | - | - | 9 | 0 | 0 | - |

Model 7: Character Independent Diversification (2 Hidden States)

Diversification Parameters:

|  | Small A | Medium A | Large A | Small B | Medium B | Large B |
| --- | --- | --- | --- | --- | --- | --- |
| Turnover | 1 | 1 | 1 | 2 | 2 | 2 |
| Extinction Fraction | 3 | 3 | 3 | 4 | 4 | 4 |

Transition Parameters:

|  | Small A | Medium A | Large A | Small B | Medium B | Large B |
| --- | --- | --- | --- | --- | --- | --- |
| Small A | - | 3 | 5 | 13 | - | - |
| Medium A | 1 | - | 6 | - | 13 | - |
| Large A | 2 | 4 | - | - | - | 13 |
| Small B | 13 | - | - | - | 9 | 11 |
| Medium B | - | 13 | - | 7 | - | 12 |
| Large B | - | - | 13 | 8 | 10 | - |

Model 8: Character Independent Diversification (3 Hidden States)

Diversification Parameters:

|  | Small A | Medium A | Large A | Small B | Medium B | Large B | Small C | Medium C | Large C |
| --- | --- | --- | --- | --- | --- | --- | --- | --- | --- |
| Turnover | 1 | 1 | 1 | 2 | 2 | 2 | 3 | 3 | 3 |
| Extinction Fraction | 4 | 4 | 4 | 5 | 5 | 5 | 6 | 6 | 6 |

Transition Parameters:

|  | Small A | Medium A | Large A | Small B | Medium B | Large B | Small C | Medium C | Large C |
| --- | --- | --- | --- | --- | --- | --- | --- | --- | --- |
| Small A | - | 3 | 5 | 19 | - | - | 19 | - | - |
| Medium A | 1 | - | 6 | - | 19 | - | - | 19 | - |
| Large A | 2 | 4 | - | - | - | 19 | - | - | 19 |
| Small B | 19 | - | - | - | 9 | 11 | 19 | - | - |
| Medium B | - | 19 | - | 7 | - | 12 | - | 19 | - |
| Large B | - | - | 19 | 8 | 10 | - | - | - | 19 |
| Small C | 19 | - | - | 19 | - | - | - | 15 | 17 |
| Medium C | - | 19 | - | - | 19 | - | 13 | - | 18 |
| Large C | - | - | 19 | - | - | 19 | 14 | 16 | - |

The optimization of each model was performed using 100 random starting points, and the run with the highest likelihood was used to rank models using AIC. The results of model testing are shown in the tables below for both dietary ecotype and size. We used the weighted average (ωAIC) of all models, respectively, to estimate the tip associated diversification rates.

**Table 1.** Ranked MuHiSSE results for body size and ecotype, in descending order of support for tested models.

| **Body Size Model** | **Brief Description** | **Free Parameters** | **Liklihood** | **AIC** | **𝝙AIC** | **ωAIC** |
| --- | --- | --- | --- | --- | --- | --- |
| MuHiSSE Relaxed | Relaxed state dependent diversification with a hidden state | 26 | -1414.42 | 2875.98 | 0.00 | 0.57 |
| Large Absorbing | No transitions out of large state | 21 | -1417.00 | 2876.59 | 0.60 | 0.42 |
| MuHiSSE | State dependent diversification with a hidden state | 25 | -1419.30 | 2883.46 | 7.48 | 0.01 |
| Small Absorbing | No transitions out of small state | 21 | -1428.61 | 2899.82 | 23.84 | 0.00 |
| CID2 | Character-independent with 2 hidden states | 17 | -1441.98 | 2904.62 | 28.63 | 0.00 |
| MuSSE | State dependent diversification | 12 | -1442.17 | 2909.28 | 33.30 | 0.00 |
| "Dull" | Equal rates | 7 | -1447.66 | 2911.75 | 35.77 | 0.00 |
| CID3 | Character-independent with 3 hidden states | 19 | -1440.25 | 2914.16 | 38.17 | 0.00 |

| **EcotypeModel** | **Brief Description** | **Free Parameters** | **Liklihood** | **AIC** | **𝝙AIC** | **ωAIC** |
| --- | --- | --- | --- | --- | --- | --- |
| MuHiSSE Relaxed | Relaxed state dependent diversification with a hidden state | 26 | -1414.42 | 2875.98 | 0.00 | 0.57 |
| Large Absorbing | No transitions out of large state | 21 | -1417.00 | 2876.59 | 0.60 | 0.42 |
| MuHiSSE | State dependent diversification with a hidden state | 25 | -1419.30 | 2883.46 | 7.48 | 0.01 |
| Small Absorbing | No transitions out of small state | 21 | -1428.61 | 2899.82 | 23.84 | 0.00 |
| CID2 | Character-independent with 2 hidden states | 17 | -1441.98 | 2904.62 | 28.63 | 0.00 |
| MuSSE | State dependent diversification | 12 | -1442.17 | 2909.28 | 33.30 | 0.00 |
| "Dull" | Equal rates | 7 | -1447.66 | 2911.75 | 35.77 | 0.00 |
| CID3 | Character-independent with 3 hidden states | 19 | -1440.25 | 2914.16 | 38.17 | 0.00 |
