# Supplementary material for: Phylogeny of the damselfishes (Pomacentridae) and patterns of asymmetrical diversification in body size and feeding ecology
Source: PLoS One. 2021 Oct 27;16(10):e0258889. doi: 10.1371/journal.pone.0258889 (PMC8550381; doi:10.1371/journal.pone.0258889)
Supplement: S1 File — (ZIP) [file pone.0258889.s001.zip › SupportingInfoFinal/S2_Table_Primers.docx]

**Supplemental Table 1** Primers and primer sequences used for amplification and cycle sequencing in this study. *Best fit nucleotide substitution model, based on Akaike information criteria score, for each gene region was determined using PartitionFinder (Lanfear et al. 2016). **Gene regions retrieved exclusively from GenBank.

| **Gene** | **Primer** | **Primer sequence** | **Model of nucleotide substitution*** |
| --- | --- | --- | --- |
| *12S rDNA* | 12S53F  12S489F  12S613R  12S991R | CAC AAA GGC TTG GTC CTG ACT TT  CTG GGA TTA GAT ACC CCA CTA TGC  TCG GTT CTA GAA CAG GCT CCT CTA G  GGT ACA CTT ACC ATG TTA CGA CT | GTR + I + G |
| *16S rDNA* | 16AR  16BR | CGC CTG TTT ATC AAA AAC AT  CCG GTC TGA ACT CAG ATC ACG T | GTR + I + G |
| *rag2* | RAG2-f1  RAG2-r2 | GAG GGC CAT CTC CTT CTC CAA  GTC TGT AGA GTC TCA CAG GAG AGC A | K80 + I + G |
| *bmp4* | Bmp4-2Fa  Bmp4-2Fb  Bmp4-2R | TCT YAT YTC AGA GCA CAT GGA GAG G  AAC CTC ACC AGC ATT CCA GA  ATC GCT GAA GTC CAC GTA CA | GTR + I + G |
| *rag1* | F1a  F420  F937  Q1F  Q9R  R670  Q1R | GGC CGC CAG ATC TTY CAG CC  GTC TGC GAG GAC AGA GAC AGA CAT A  GAC ATC GGG AAC GCC AGC GAR TT  AGC TGT AGT CAG TAY CAC AAR ATG  GTG TAG AGC CAG TGR TGY TT  GAY GAG AAG ATG GTG CGT GAG ATG G  AGC TGT AGT CAG TAY CAC AAR ATG | K80 + I + G |
| *ND3* | F270  R750  F280 | TAY RTM TCM ATC TAC TGA TGA GG  TTG ATT TCG RCTC AAA ARW TTR TGG  GAT GAG GMT CWT AAT CTT TCT AGT A | GTR + I + G |
| *ATP*** |  |  | GTR + I + G |
| *COI*** |  |  | GTR + I + G |
| *cytB*** |  |  | GTR + I + G |
| *tmo4c4* | Tmo-f1-5  Tmo-f1-6  Tmo-r1-3 | CCT CCG GCC TTC CTA AAA CCT CTC  GAA AAG AGT GTT TGA AAA TGA  CAT CGT GCT CCT GGG TGA CAA AGT | K80 + I + G |
| *DLX2* | F760  R2 | GAA GAG AGY GAG CCA GAA ATC  AGT TTG CCA AAA ACG ACG AA | K80 + I + G |
