# Supplementary figures and images for: Phylogeny of the damselfishes (Pomacentridae) and patterns of asymmetrical diversification in body size and feeding ecology
Source: PLoS One. 2021 Oct 27;16(10):e0258889. doi: 10.1371/journal.pone.0258889 (PMC8550381; doi:10.1371/journal.pone.0258889)

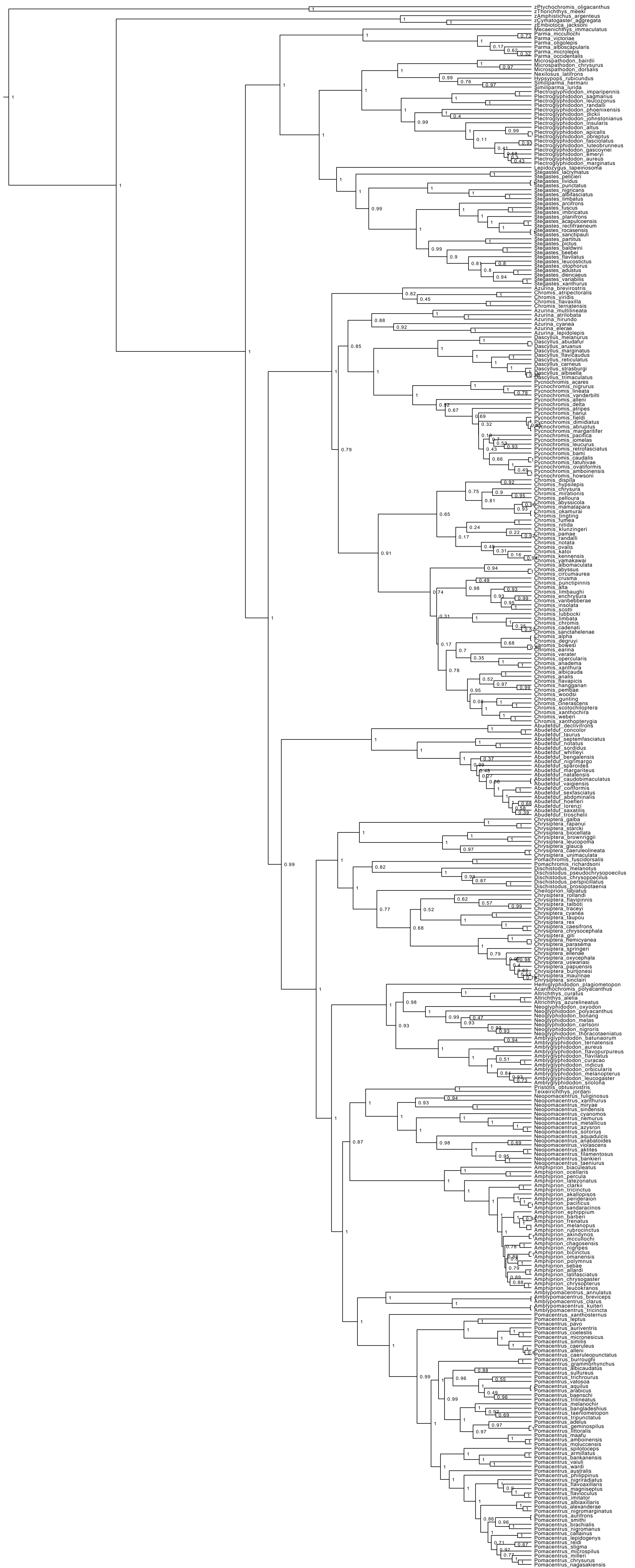

Supplement: S1 File — (ZIP) [file pone.0258889.s001.zip › SupportingInfoFinal/S2_Fig_TreeSupport.pdf]

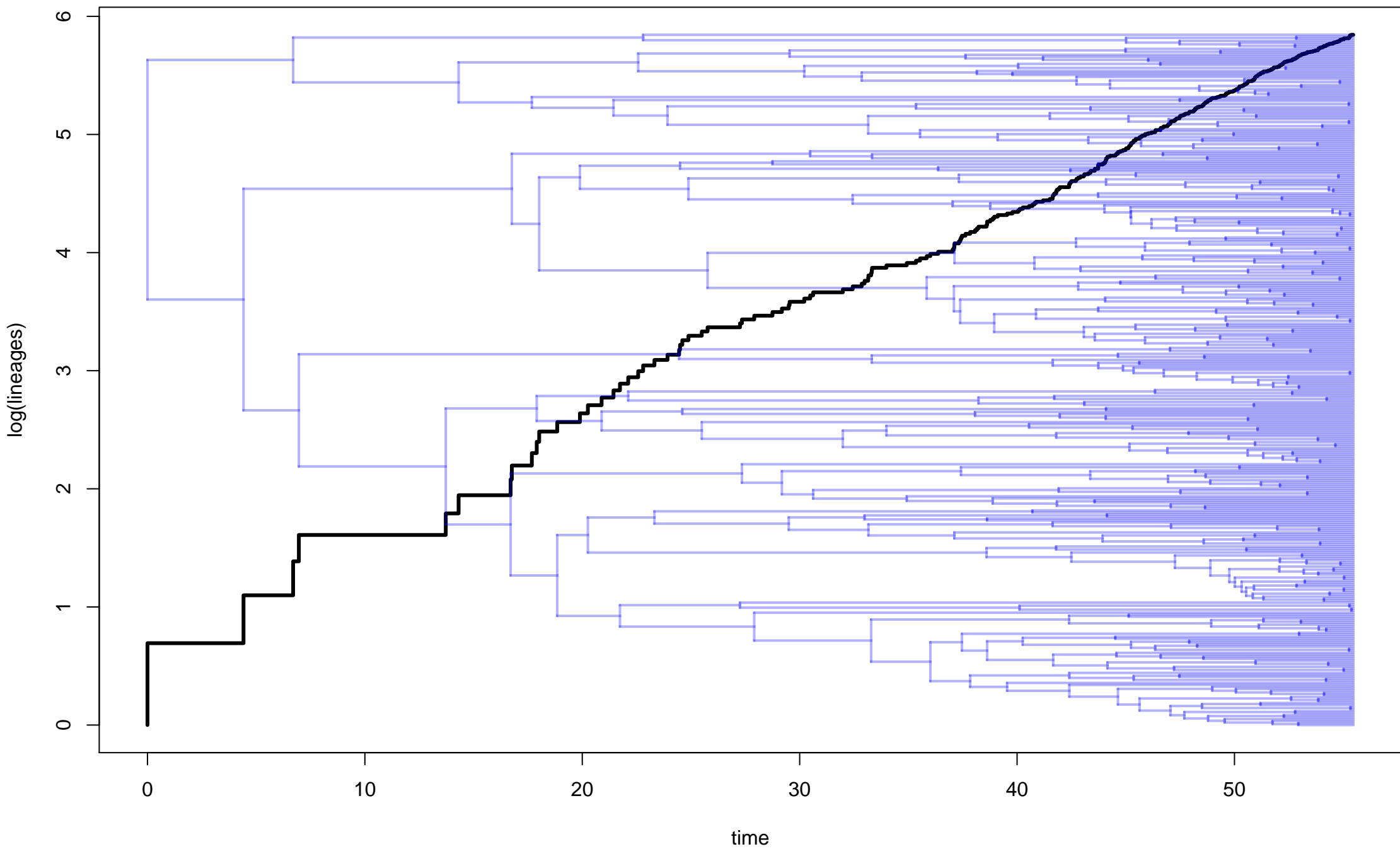

Supplement: S1 File — (ZIP) [file pone.0258889.s001.zip › SupportingInfoFinal/S4_Fig_LTT.pdf]

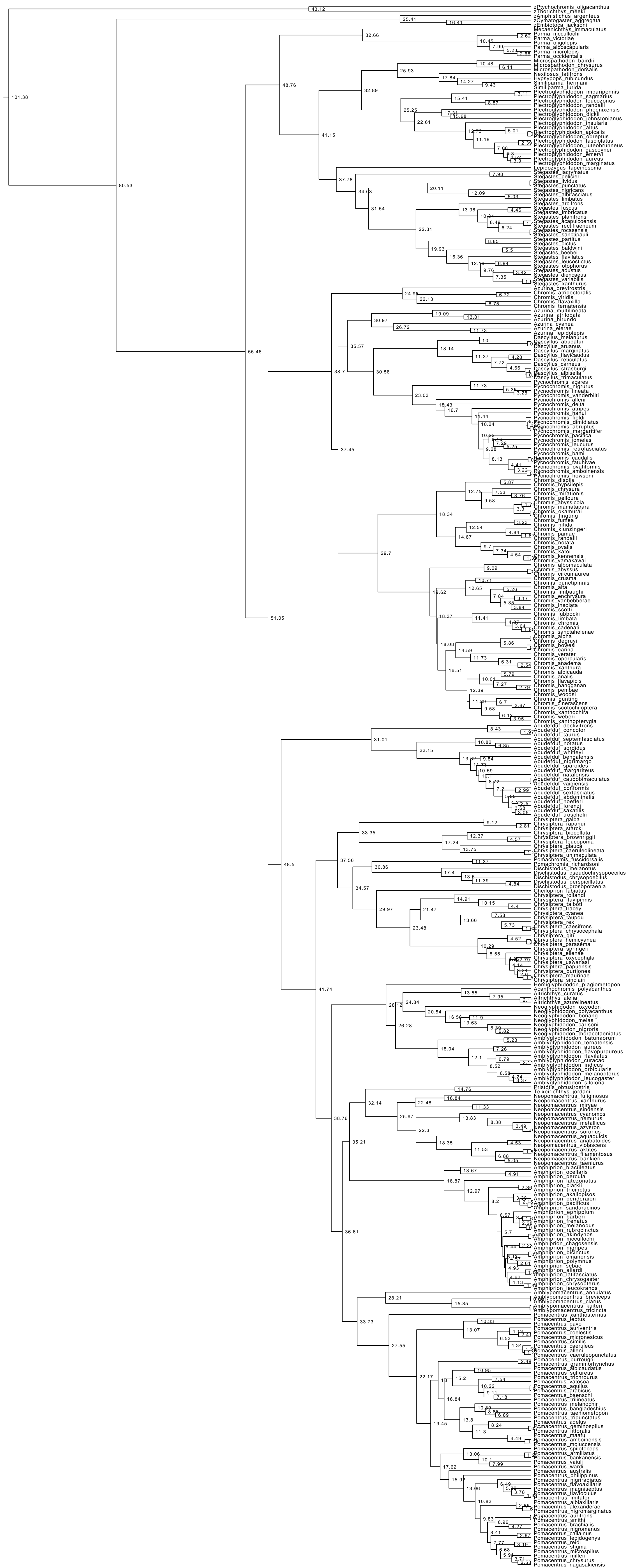

Supplement: S1 File — (ZIP) [file pone.0258889.s001.zip › SupportingInfoFinal/S3_Fig_TreeAges.pdf]
